# A Mechanism for Genome Size Reduction Following Genomic Rearrangements

**DOI:** 10.3389/fgene.2018.00454

**Published:** 2018-10-09

**Authors:** Longhui Ren, Wei Huang, Ethalinda K. S. Cannon, David J. Bertioli, Steven B. Cannon

**Affiliations:** ^1^Interdepartmental Genetics Graduate Program, Iowa State University, Ames, IA, United States; ^2^Department of Agronomy, Iowa State University, Ames, IA, United States; ^3^Department of Computer Science, Iowa State University, Ames, IA, United States; ^4^Institute of Biological Sciences, University of Brasiìlia, Brasiìlia, Brazil; ^5^Center for Applied Genetic Technologies, University of Georgia, Athens, GA, United States; ^6^Corn Insects and Crop Genetics Research Unit, United States Department of Agriculture–Agricultural Research Service, Ames, IA, United States

**Keywords:** genome size evolution, recombination, genomic rearrangement, sequence removal, transposable element

## Abstract

The factors behind genome size evolution have been of great interest, considering that eukaryotic genomes vary in size by more than three orders of magnitude. Using a model of two wild peanut relatives, *Arachis duranensis* and *Arachis ipaensis*, in which one genome experienced large rearrangements, we find that the main determinant in genome size reduction is a set of inversions that occurred in *A. duranensis*, and subsequent net sequence removal in the inverted regions. We observe a general pattern in which sequence is lost more rapidly at newly distal (telomeric) regions than it is gained at newly proximal (pericentromeric) regions – resulting in net sequence loss in the inverted regions. The major driver of this process is recombination, determined by the chromosomal location. Any type of genomic rearrangement that exposes proximal regions to higher recombination rates can cause genome size reduction by this mechanism. In comparisons between *A. duranensis* and *A. ipaensis*, we find that the inversions all occurred in *A. duranensis*. Sequence loss in those regions was primarily due to removal of transposable elements. Illegitimate recombination is likely the major mechanism responsible for the sequence removal, rather than unequal intrastrand recombination. We also measure the relative rate of genome size reduction in these two *Arachis* diploids. We also test our model in other plant species and find that it applies in all cases examined, suggesting our model is widely applicable.

## Introduction

Genome size varies extensively in eukaryotes, and the variation is still tremendous when we only look at plants (Ohri, 1998; Bennett et al., 2000; Bennett and Leitch, 2005, 2011; Hendrix and Stewart, 2005; Price et al., 2005; Ammiraju et al., 2006; Gregory et al., 2007; Michael, 2014). For example, *Arabidopsis thaliana* has a genome size of ∼135 Mbp (Arabidopsis Genome Initiative, 2000) whereas the genome size of *Allium cepa* is ∼16,000 Mbp (Arumuganathan and Earle, 1991; Ricroch et al., 2005). The most extreme known plant genome sizes are 61 Mbp for *Genlisea tuberosa* (Fleischmann et al., 2014) and 150,000 Mbp for *Paris japonica* (Pellicer et al., 2010) – a 2,459-fold difference. What mechanisms explain the vast difference in genome sizes in eukaryotes – sometimes with order-of-magnitude changes even within a single genus? It is believed that genome size is affected by several factors, including polyploidization, transposable element (TE) proliferation and deletion, and other types of sequence insertions and deletions (Vicient et al., 1999; Rabinowicz, 2000; Petrov, 2001; Bennetzen, 2002; Devos et al., 2002; Vitte and Panaud, 2003, 2005; Ma et al., 2004; Adams and Wendel, 2005; Bennetzen et al., 2005; Hawkins et al., 2006; Neumann et al., 2006; Piegu et al., 2006; Grover and Wendel, 2010; Chenais et al., 2012; Michael, 2014; Soltis et al., 2015). Genomic rearrangements, including inversions, translocations, fusions, and fissions, can change the chromosomal architecture dramatically. They are common during the evolution of plants, and plant genomes have generally experienced more rapid chromosomal architecture changes than mammalian genomes (Salse et al., 2009). Do genomic rearrangements have effects on genome size evolution in plants? If they do, what is the pattern of genome size changes after the rearrangements? What are the primary drivers behind the changes?

Previous analyses of *Arabidopsis thaliana* and *Arabidopsis lyrata* have shown that genomic rearrangements are associated with genome shrinkage in *A. thaliana* (Hu et al., 2011). However, it is not known what mechanisms are responsible for genome size changes following genomic rearrangements. The genome assemblies of two wild ancestors of cultivated peanut, *Arachis duranensis* (*Ad*) and *Arachis ipaensis* (*Ai*), which separated from each other about two million years ago, provide useful models of genomic evolution. Several large inversions occurred since the divergence of these species, and they also differ substantially in genome size (Bertioli et al., 2016). This provides an opportunity to examine genomic changes in both inverted and non-inverted regions, and in chromosomes with and without inversions.

We propose a model for genome size changes related to genomic rearrangements, and investigate the underlying mechanism in these two *Arachis* diploids. We also test the model and mechanisms in other closely related plant genomes to determine whether these mechanisms are widespread in plant genomes. This research provides new insights into the relationship between genomic rearrangements and genome size evolution in plants.

## Materials and Methods

### Genome Assembly and Annotation Access

Genome assemblies and annotations used in this study are publicly available online. Genome assemblies and annotations of *Ai* and *Ad* are available on PeanutBase^1^ website. Genome assembly and annotation of *Vigna radiata* is available on Legume Information System^2^ website. Genome assemblies and annotations of *Glycine max*, *Phaseolus vulgaris*, *Sorghum bicolor*, *Setaria italica*, *Brachypodium distachyon*, and *Zea mays* are available from PhytozomeV10^3^.

### Dot-Plot Visualization of Synteny

Dot-plot comparisons between *Ai* and *Ad* were made using mummer and mummerplot from the MUMmer suite of alignment tools (Kurtz et al., 2004). Dot-plot comparisons between other species pairs in the validation part were generated using DAGchainer and Java package XY-plot included in DAGchainer (Haas et al., 2004).

### Gene Density Difference Calculation

Gene density difference was calculated by first dividing each chromosome into 500 partitions equally in both two *Arachis* species, then every partition in one genome has a corresponding partition in the other genome and they form a pair. The average number of genes in 100 kb was calculated as the gene density in each partition. The gene density difference was calculated by subtracting the gene density of *Ad* from that of *Ai* for each pair of partitions.

### Syntenic Blocks and Size Ratio Calculation

For *Ai* and *Ad*, and also for species pairs in the validation part, the peptide sequences were used to perform the synteny analysis. BLAST was used to perform the search for homologous sequence pairs within each of those species pairs with *e* value ≤ 1 × 10^−10^ (Camacho et al., 2009). After getting the blast result, the top hit of each query sequence was selected. Synteny was calculated for each of those species pairs using DAGchainer (Haas et al., 2004). Syntenic blocks resulted from the DAGchainer were manually checked, and small overlapping or misplacing syntenic blocks were removed. Size ratios of syntenic blocks between *Ai* and *Ad* were calculated by dividing the size of syntenic block in *Ai* by the size of corresponding syntenic block in *Ad*. The overall size ratios of syntenic blocks in inverted and non-inverted regions were calculated by excluding chromosomes 7 and 8. Size ratios of syntenic blocks in the validation part were calculated using these comparisons: *Vigna radiata*/*Phaseolus vulgaris*, *Sorghum bicolor*/*Setaria italica,* and *Sorghum bicolor*/*Zea mays*.

### Transposable Elements Identification

Transposable elements were identified for *Ai* and *Ad* using RepeatMasker^4^. The database used to identify TEs is the combination of Repbase (version 20150807) and *Arachis* repeat libraries (mobile-elements-BB051914.fa and mobile-elements-AA051914.fa) which are available at PeanutBase (see footnote 1). The number of TEs was counted for each of the syntenic blocks in the two *Arachis* species, in order to calculate the ratio of TE numbers between corresponding syntenic blocks. TEs identified were categorized into different components based on the result from RepeatMasker. The TE components of inverted and non-inverted regions were counted by excluding chromosomes 7 and 8.

### Local Gene Duplication Identification

The protein sequences of pre-calculated gene families in angiosperms were downloaded from Phytozome 10. For each gene family, multiple sequence alignments (MSAs) were generated using Muscle (Edgar, 2004). Hidden Markov Models (HMMs) were built from the alignment of each gene family and were used to search against the protein sequences of *Ai* and *Ad,* respectively, using HMMER (Eddy, 1998). Genes in two *Arachis* species were assigned to gene families based on their best hits. Local gene duplication was defined as genes from the same gene family within 10 successive genes, and it was calculated by sliding window method with a window size of 10 genes and a step of 1 gene. Locally duplicated gene was recorded for each window and was used to count the number of local gene duplications. With the exclusion of chromosomes 7 and 8, the total number of local gene duplications in inverted and non-inverted regions was counted in two *Arachis* species, and the ratio of local gene duplications was calculated based on that.

### Sequence Alignment and Characterization

The inversion at the end of chromosome 1 of *Ai* and *Ad* was chosen to perform the sequence alignment. The sequence alignment was built on the DNA sequences of the chosen inversion using MAUVE (Darling et al., 2010). The alignment was built using default parameters in MAUVE, except for setting min LCB weight as 30. Unaligned sequences, which are gaps in the alignment, were extracted from the result of MAUVE. The total length of unaligned sequences in length intervals was calculated by taking the sum of all unaligned sequences in a specific length interval. TEs identified earlier were used to perform the genomic component analysis for all unaligned sequences and unaligned sequences in length intervals. Repeats of at least 50 bp in length located within 100 base pairs of the start or end point of unaligned sequences were identified as flanking repeats.

### Gene Density and Recombination Rate Distribution

Gene density distributions of *Ai* and *Ad* were generated using CViT (Cannon and Cannon, 2011). Recombination rate distribution of *Ad* was visualized by drawing genetic distances of markers from previous research (Nagy et al., 2012) along the chromosome using CViT.

### Intact and Solo LTRs Identification

LTR retrotransposons were identified from the two genomes of *Ai* and *Ad* using LTR_FINDER (Xu and Wang, 2007). DNA sequences of 5′LTR and 3′LTR of those LTR retrotransposons were extracted and used as query sequences. Blast search of these query sequences against the whole genome sequence was performed in *Ai* and *Ad*, respectively. The blast results were filtered with full-length coverage of query sequences and 100% sequence identity. We have experimented with various sequence identity criteria (100%, 95%, and 90% sequence identity), and they all show a similar pattern. We chose to present the result under the most stringent criterion. The result from the LTR_FINDER was treated as intact LTR retrotransposons. The filtered blast hits were counted as solo LTRs.

### Relative Rate of Size Reduction Calculation

The relative rate of size reduction was calculated using the formula:

$$x=\left(1-\sqrt[{n}]{b/a}\right)\times 100$$

*a*: length of inverted region in *Ai*; *b*: length of inverted region in *Ad*; *n*: number of generations after the inversion occurred; *x*: relative rate of size reduction per 100 base pairs per generation. The lengths of inverted regions in two *Arachis* species were determined by the synteny calculated earlier between these two species.

## Results

### Inversions Occurred in *Arachis duranensis* After Speciation With *Arachis ipaensis*

**FIGURE 1:**
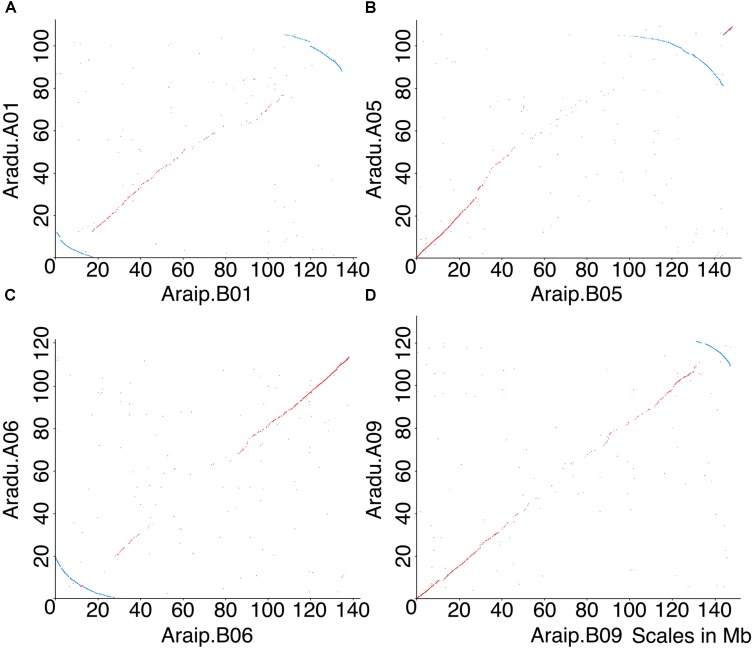
Dot-plot comparisons of chromosomes between *Ai* and *Ad* reveal five inversions. *X*-axis represents chromosomal position in *Ai*, and *y*-axis represents chromosomal position in *Ad*. Chromosomes in *Ad* are named as A01, A02, etc., and chromosomes in *Ai* are named as B01, B02, etc. Forward matches are shown in red, while reverse matches are shown in blue. **(A)** Dot-plot comparison of chromosome B01 and A01, blue arc indicates inversion between two genomes. **(B)** Dot-plot comparison of chromosome B05 and A05, blue arc indicates inversion between two genomes. **(C)** Dot-plot comparison of chromosome B06 and A06, blue arc indicates inversion between two genomes. **(D)** Dot-plot comparison of chromosome B09 and A09, blue arc indicates inversion between two genomes. Chromosomal pseudomolecules were given numbers corresponding to genetic linkage maps in Bertioli et al. (2016), which mostly do not have known correspondences with cytogenetic chromosome assignments.

Several inversions are evident between *Arachis duranensis* and *Arachis ipaensis* (**Figure 1**) (Bertioli et al., 2016), but we wished to determine the species in which inversions occurred. This could, in principle, be accomplished by comparing the synteny plots between the two species involved and a third species. However, the other sequenced legume genomes [e.g., *Glycine max* (soybean), *Phaseolus vulgaris* (common bean), or *Medicago truncatula* (barrel medic)] are all separated from *Ai* and *Ad* by ∼58 million years (Lavin et al., 2005) – a sufficiently long separation that dot-plot comparisons don’t definitively show which of the *Arachis* species had any particular inversion. However, a collection of circumstantial evidence supports that the genomic rearrangements occurred in *Ad* rather than in *Ai*. The key evidence is in disrupted gene density gradients, coincident with inversion breakpoints.

**FIGURE 2:**
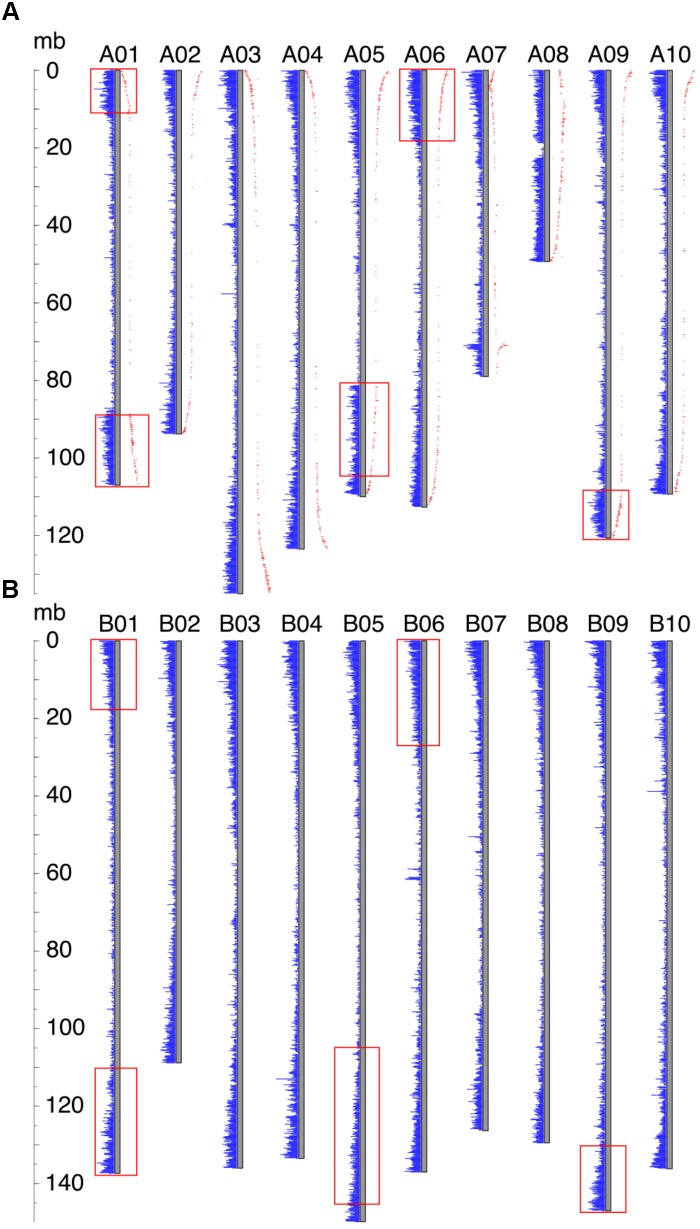
Gene density distribution and genetic distances along the chromosome. Each gray bar represents a chromosome, and gene densities are represented by blue bars. The ruler on the left side indicates the chromosomal position. **(A)** Gene density distribution and genetic distances of *Ad*. Genetic distance is shown in red dots, and the distance from the gray bar to the red dot indicates the value of the genetic distance. Inverted regions are highlighted by red rectangles. **(B)** Gene density distribution of *Ai*. Regions in *Ai* which correspond to the inverted regions in *Ad* are highlighted by red rectangles.

**FIGURE 3:**
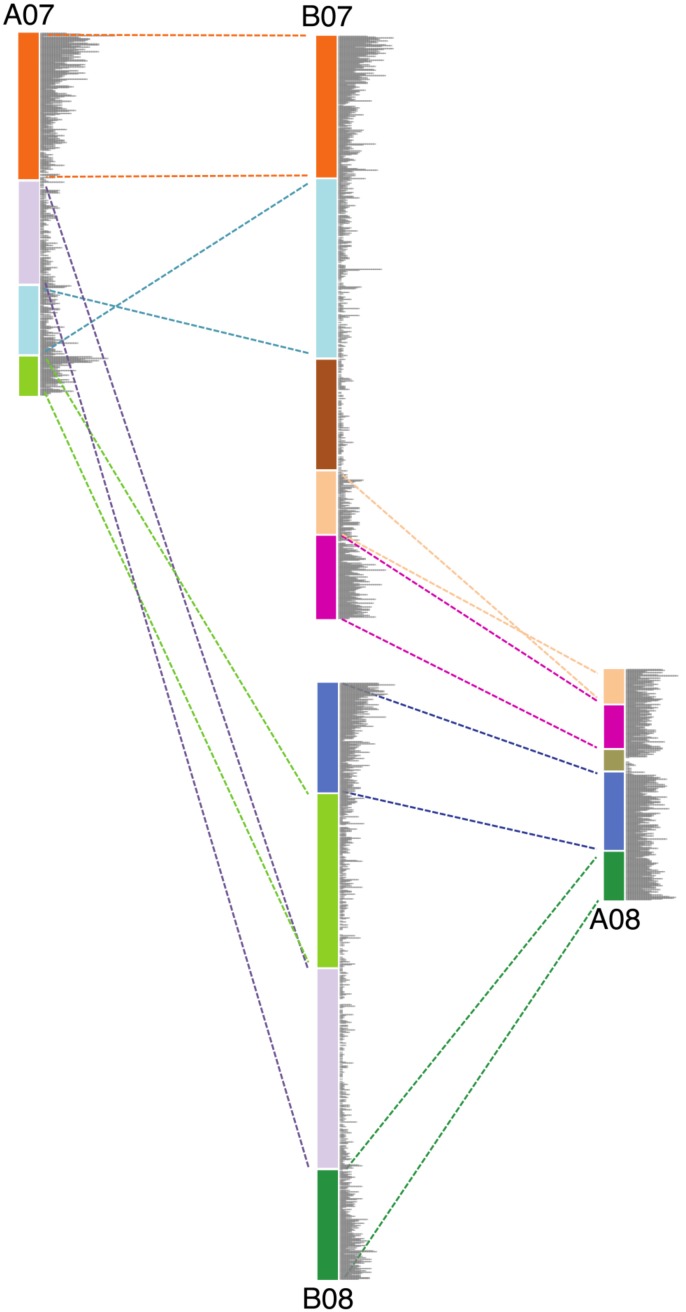
Schematic diagram showing the genomic rearrangements between chromosomes 7 and 8 in *Ai* and *Ad*. Blocks in same color represent syntenic genomic segments, and the dashed lines in the same color indicate the orientation of those segments. Gray bars represent the gene density.

For all *Ai* chromosomes, there is a gene density gradient, rising smoothly from low densities at chromosome centers to high density near the telomeres, giving a U-shaped density plot (**Figure 2**). This U-shaped density plot is also present in about half of the *Ad* chromosomes, but this pattern is disrupted in chromosomes showing an inversion between *Ai* and *Ad* – and critically, the pattern is disrupted only in the *Ad* chromosomes (**Figure 2**). The unusual distributions of gene density in the inverted regions in *Ad* are also evident in the gene density differences between the two *Arachis* species (**Supplementary Figure 1**). The unusual density differences evident between *Ad* and *Ai* and coincident with regions with inversions between the two genomes, are consistent with *Ai* having the ancestral state (relative to the progenitor of the two species), and all inversions occurring in *Ad*. The genome size of *Ad* is smaller than that of *Ai*, and the chromosomal differences are greatest for chromosomes with inversions (**Table 1**). Chromosome pair 7 and 8 also have large size differences because several complex genomic rearrangements have occurred in and between these two chromosome pairs (**Figure 3**) (Bertioli et al., 2016).

**Table 1:** Comparison of chromosome size in *Ai* and *Ad.*

*A. ipaensis*		*A. duranensis*		*A. ipaensis* size/ *A. duranensis* size
Chrom	Size (bp)	Chrom	Size (bp)	
B01	137,414,913	A01	107,035,537	1.28
B02	108,997,779	A02	93,869,048	1.16
B03	136,109,863	A03	135,057,546	1.01
B04	133,615,181	A04	123,556,382	1.08
B05	149,900,536	A05	110,037,037	1.36
B06	137,147,148	A06	112,752,717	1.22
B07	126,351,151	A07	79,126,724	1.60^a^
B08	129,606,920	A08	49,462,234	2.62^a^
B09	147,089,397	A09	120,672,674	1.22
B10	136,175,642	A10	109,463,236	1.24

*^a^Chromosome pairs 7 and 8 have experienced several genomic rearrangements.*

### Model of Genome Size Reduction

**FIGURE 4:**
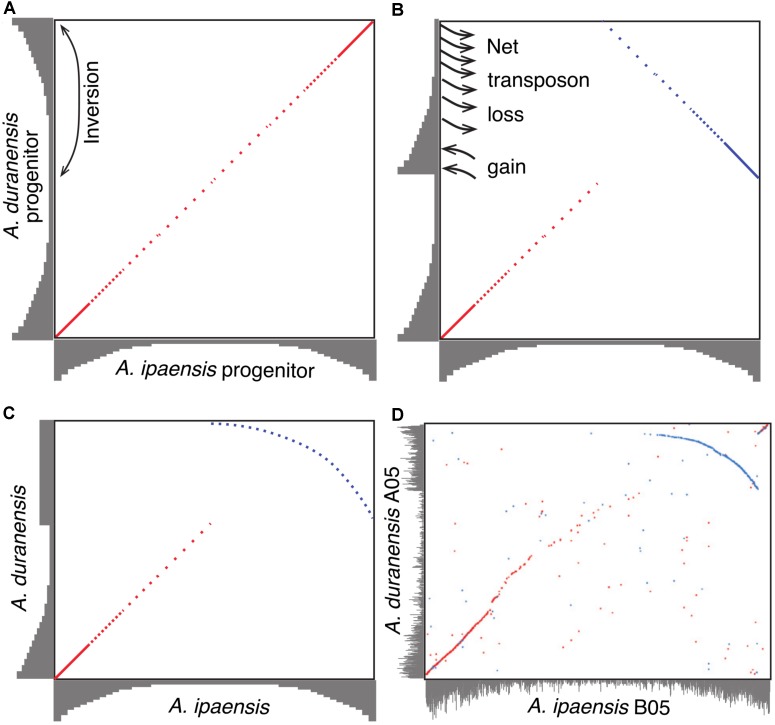
Model of genome size reduction of inverted regions in *Ad*. Gene densities are shown in gray. Forward matches are shown in red, while reverse matches are shown in blue. **(A)** *Ai* and *Ad* share the same distribution of gene densities right after the divergence of the two species. Inversion brings the repeat-rich gene-poor proximal region to distal region, and gene-rich repeat-poor distal region to proximal region. **(B)** The repeat-rich region starts to loss DNA content via the deletion of repeats driven by recombination after becoming distal region, whereas the repeat-poor region begins to gain DNA content via the accumulation of repeats. **(C)** Higher rate of repeat deletion causes the size reduction of inverted region, and shapes the plot of the inverted region into this characteristic arc. **(D)** Dot-plot comparison between chromosome B05 and A05 showing that characteristic arc.

A model to explain the relationship between inversions and genome size changes was suggested in Bertioli et al. (2016) (**Figure 4**). In this paper, we flesh out that model by examining potential of sequence loss and gain mechanisms, and test the predictions in other species. The model, illustrated with *Ai* and *Ad*, is as follows. Immediately following divergence of these two species, they would have shared the same gradients for gene and repetitive DNA, with higher gene density (lower density of repetitive DNA) at distal (telomeric) regions and lower gene density (higher density of repetitive DNA) at proximal (pericentromeric) regions (**Figure 4A**). Inversions in *Ad* chromosomes flipped these gradients by making distal (telomeric) regions proximal (pericentromeric) region, or vice versa (**Figures 4A,B**). When a formerly proximal (pericentromeric) region became distal (telomeric) after an inversion, then it was exposed to higher recombination rates, which quickly squeezed out TEs enriched in what had been a proximal (pericentromeric) region (**Figures 4B,C**). At the same time, the formerly distal region moved inside (proximal/pericentromeric), where it slowly accumulates TEs, due to lower recombination rates in the proximal (pericentromeric) environment (**Figures 4B,C**). The process of TE removal in the newly distal/telomeric regions is much faster than the process of TE accumulation in the center, which leads to the net size reduction of the inverted region in *Ad*. These two processes affected the gradients of gene and repetitive DNA simultaneously over time and re-shaped the plot in the inverted region between these two species into a characteristic arc (**Figure 4C**). The synteny plot in the inverted region between two *Arachis* species shows the pattern predicted by the model (**Figure 4D**). This similarity suggests that this model of genome size reduction is reasonable, but empirical details are needed in order to determine mechanics: what types of sequences are removed from distal/telomeric regions? What is added in proximal/pericentromeric regions? At what relative rates?

### Changes in Size Ratio of Syntenic Blocks

**FIGURE 5:**
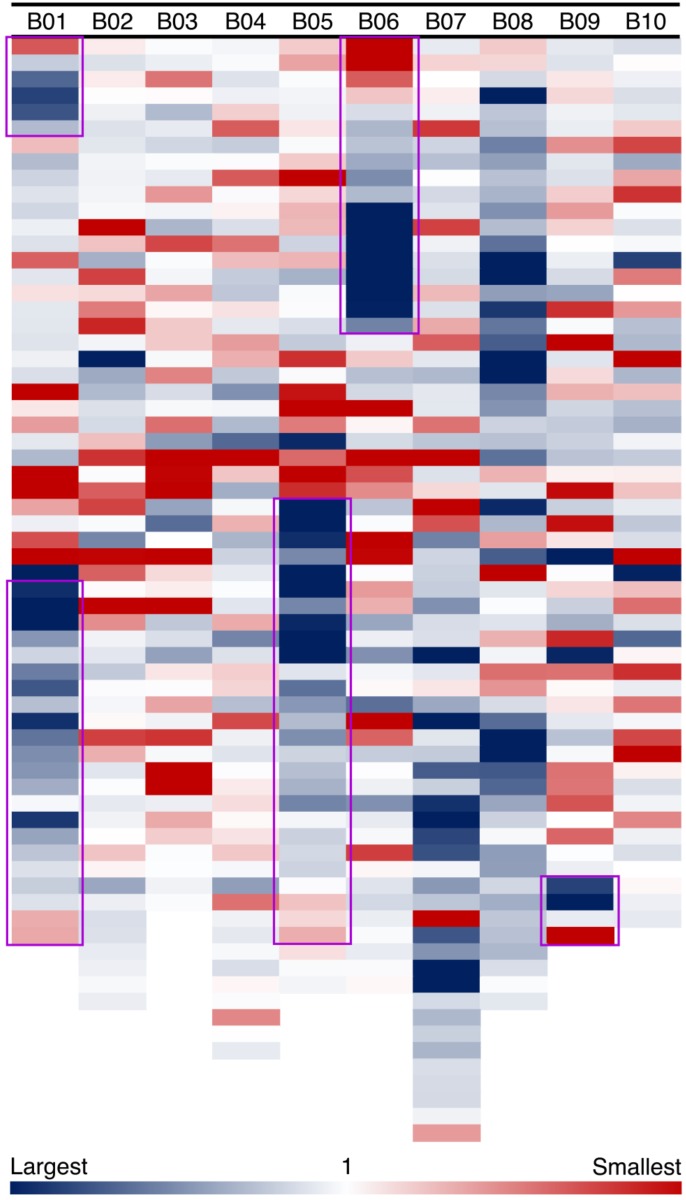
Size ratios of syntenic blocks between *Ai* and *Ad*. Each column shows a chromosome in *A. ipaensis*, and each colored block represents a syntenic block in the order of genomic position in *A. ipaensis*. Regions highlighted by purple rectangles are inverted regions in *A. duranensis*, which means those regions are flipped over in *A. duranensis*. The size ratios are calculated as *A. ipaensis*/*A. duranensis*, and are shown in the blue-white-red gradient. Blue color indicates that the ratio is larger than 1, which means the syntenic block is smaller in *A. duranensis*. Red color indicates that the ratio is smaller than 1, which means the syntenic block is larger in *A. duranensis*. White color indicates that the ratio is 1, which means the syntenic blocks have the same size.

In our model of genome size reduction, there is a particular pattern of genome size changes after an inversion: not only do the inverted regions become smaller, but they do so following a distance-dependent gradient. We measure amounts of sequence loss or gain by examining syntenic blocks across the large inversions between *Ad* and *Ai*. For the five major inversions, syntenic blocks in the newly distal (telomeric) regions in *Ad* are smaller than corresponding syntenic blocks in the proximal regions in *Ai*, whereas syntenic blocks in the proximal regions in *Ad* are larger than corresponding syntenic blocks in the distal regions in *Ai* (**Figure 5**). The distance-dependent gradients in the inverted regions are made evident by the increase in size ratios of syntenic blocks from the proximal end to the distal end in *Ad* (**Figure 5** and **Supplementary Data 1**). Additionally, most of those syntenic blocks inside the inverted regions are smaller in *Ad*, and only syntenic blocks at the very end of the newly proximal (pericentromeric) regions are larger in *Ad* (**Figure 5**). The overall size ratios of syntenic blocks (*Ai*/*Ad*) for inverted and non-inverted regions are 1.40 and 1.06, respectively, which demonstrates that the inverted regions are smaller in *Ad* while the non-inverted regions have remained approximately the same size in these two *Arachis* species.

### Transposable Elements and Local Gene Duplication

**FIGURE 6:**
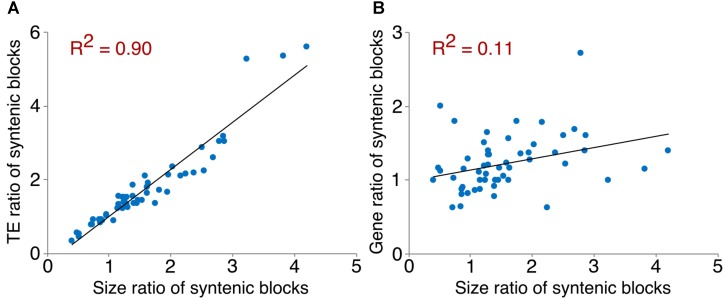
Correlation between TE/gene ratios and size ratios of syntenic blocks. An example of scatter plots showing the correlation between TE/gene ratios and size ratios of syntenic blocks on chromosome 1. **(A)** Correlation between TE ratios and size ratios of syntenic blocks on chromosome 1. **(B)** Correlation between gene ratios and size ratios of syntenic blocks on chromosome 1.

**FIGURE 7:**
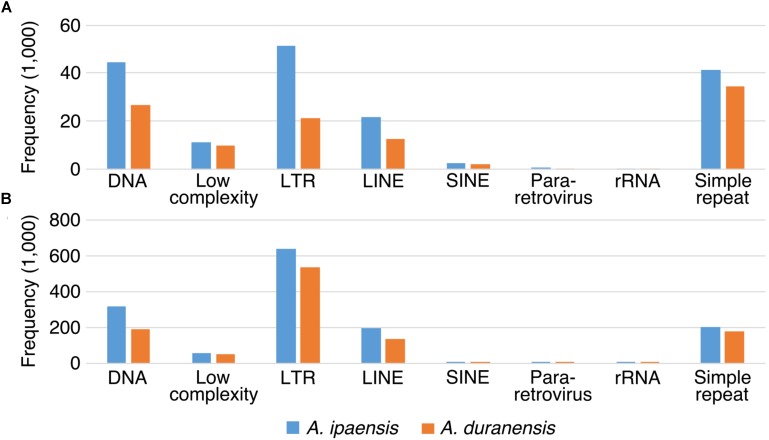
TE components of inverted and non-inverted regions. *X*-axis is the name of TE components, and *y*-axis is the frequency of TE components. TE components in *Ai* genome are in blue bars, whereas TE components in *Ad* genome are in orange bars. **(A)** TE components of inverted regions. **(B)** TE components of non-inverted regions.

We examined TEs in the genomes of these two *Arachis* species in order to better understand the nature of changes following large inversions. For chromosomes with inversions, the ratios of TE numbers in corresponding syntenic blocks are highly correlated with the size ratios of those syntenic blocks, with average *R*^2^ of 0.94 across all blocks; whereas the correlation between the gene number ratios of corresponding syntenic blocks and the size ratios of those syntenic blocks are weak, with average *R*^2^ of 0.21 across all blocks (**Figure 6** and **Supplementary Table 1**). This confirms that the change in TE content is the major reason for genome size reduction in the inverted regions. Besides the change in numbers, the TE composition was also altered in the inverted regions. Most TE components in *Ai* and *Ad* show similar patterns in inverted and non-inverted regions, although LTR elements show dramatic differences (**Figure 7** and **Table 2**). Additionally, the ratios of local gene duplications (*Ai*/*Ad*) are 1.50 and 1.27 for inverted and non-inverted regions, respectively, suggesting that changes in local gene duplications and losses also contributes to the genome size reduction after the inversion.

**Table 2:** Comparison of TEs between *Ai* and *Ad* in inverted and non-inverted regions.

Transposable elements	Inverted			Non-inverted		
	
	*Ai*	*Ad*	*Ai*/*Ad*	*Ai*	*Ad*	*Ai*/*Ad*
DNA	44697	26903	1.66	320787	193344	1.66
Low complexity	11373	9646	1.18	59088	53011	1.11
LTR	51269	21088	2.43	639715	540151	1.18
LINE	21547	12537	1.72	199677	137948	1.45
SINE	2367	1917	1.23	8468	6723	1.26
Pararetrovirus^a^	716	413	1.73	5386	1695	3.18
rRNA	19	22	0.86	112	96	1.17
Simple repeat	41227	34632	1.19	205947	178585	1.15

*^a^The ratios of pararetrovirus also show large difference, but the numbers are very small in inverted regions comparing to most TE types.*

### Length and Components of Unaligned Sequences

**FIGURE 8:**
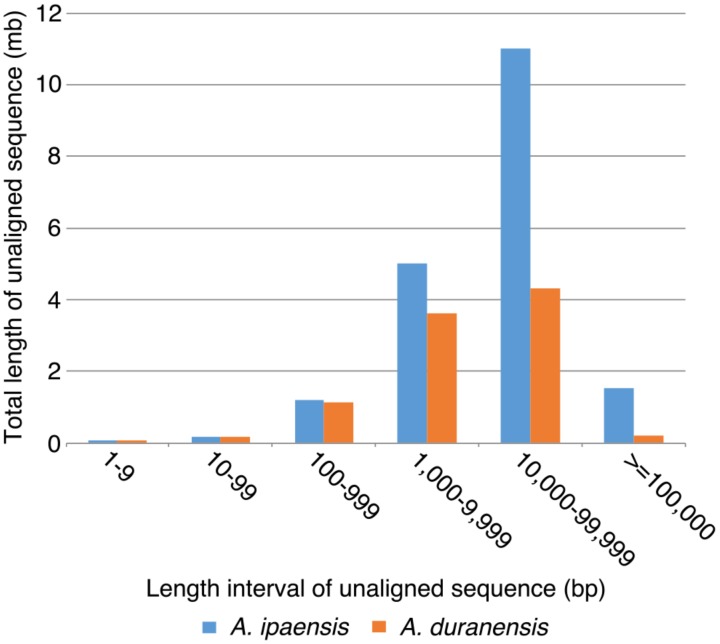
Total length of unaligned sequences in different length intervals. *X*-axis is the length intervals of unaligned sequences, and *y*-axis is the total length of unaligned sequences. Blue bars represent total length of unaligned sequences in *Ai*, whereas orange bars represent that in *Ad*.

**FIGURE 9:**
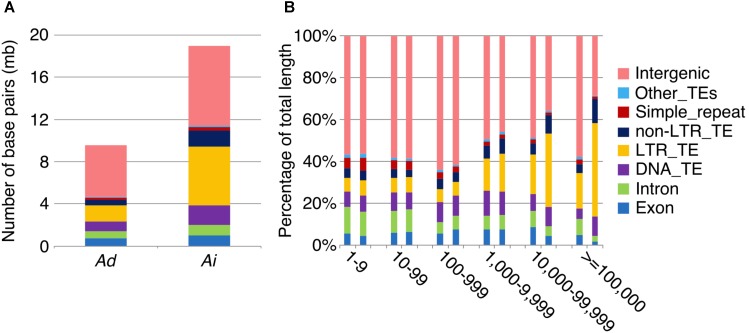
Transposable element components in unaligned sequences. Different colors represent different types of transposable elements as shown on the right. **(A)** Number of base pairs of TE components in unaligned sequences. *Ad* is on the left side, whereas *Ai* is on the right side. **(B)** TE component percentage of total sequence length in different length intervals of unaligned sequences. *X*-axis is the length interval of unaligned sequences, and *y*-axis is the percentage of total sequence length. For each length interval, *A. duranensis* is shown on the left and *A. ipaensis* is shown on the right.

To further understand the process of genome size reduction in the inverted regions, we examined a large inverted region on chromosome 1, focusing on two genomic fractions: alignable and unalignable sequence (the latter comprised of sequences between aligned sequence in the syntenic region). There is no obvious difference in the total length between two *Arachis* species for unaligned sequences shorter than 1,000 bp. However, the total length of unaligned sequences starts to show large differences in those sequences longer than 1,000 bp (**Figure 8**), suggesting that those long unaligned sequences are the main causes for the genome size reduction in the inverted regions. The count of short unaligned sequences in the two species is roughly equal (34,339 and 33,333 shorter than 1,000 bp in *Ai* and *Ad*, respectively) (**Figure 8** and **Supplementary Figure 2**). In contrast, the number of long unaligned sequences differs between the two species and significantly contributes to the size difference of the two *Arachis* species (**Figure 8** and **Supplementary Figure 2**). The unaligned sequences in two *Arachis* diploids not only differ in numbers and lengths, but also show differences in genomic components. There are more TEs in unaligned sequences of *Ai* comparing to that of *Ad*, especially the LTR elements holding the largest difference, while other genomic components appear to be relatively the same between two *Arachis* species (**Figure 9**). This observation is consistent with the above-mentioned inference that the LTR elements are the major component contributing to the genome size reduction in inverted regions. Furthermore, the percentages of genomic components remain almost the same between two *Arachis* species for shorter (<1,000 bp) unaligned sequences, but start to deviate for longer (≥1,000 bp) unaligned sequences (**Figure 9**). These results together lead to the conclusion that the genome size reduction in the inverted regions is mainly caused by the changes of TEs, especially LTR elements, in those longer unaligned sequences.

### Recombination Rate Distribution in *Ad*

In our model of genome size reduction, locationally dependent recombination is the major driver behind this process, promoting the net removal of TEs where recombination rates are highest. The model requires the recombination rate to be higher in the distal regions than the proximal regions – in other words, that chromosomal location is the independent driver of sequence change, affecting recombination rates, and therefore rates of sequence loss in high-recombination distal locations. Size reduction following a large inversion is not due to some characteristic intrinsic to the sequence; rather, the chromosomal location is most important. This pattern of location changing recombination rates following a large inversion is evident in genetic distances of markers in *Ad* from previous research (Nagy et al., 2012), displayed across all *Ad* chromosomes (**Figure 2**). In the non-inverted regions, the genetic distances increase rapidly in the distal region while remaining stable in the proximal region, indicating that the recombination rate is indeed much higher in the distal region than that of the proximal region (**Figure 2**). Most importantly, in the inverted regions, the former proximal regions, which likely had low recombination rate before the inversion, now have high recombination rate after moving into distal environments (**Figure 2**). In contrast, the formerly distal regions, which likely had high recombination rates before the inversion, now have low recombination rate after moving into proximal environments (**Figure 2**).

### Underlying Mechanisms of Genome Size Reduction

We reason that a mechanism for size reduction in inverted regions should involve recombination to remove TEs – especially LTR elements, which comprise the largest source of difference in inverted regions. Unequal intrastrand recombination and illegitimate recombination have been shown to cause deletions and to reduce genome growth (Bennetzen, 2002; Devos et al., 2002; Ma et al., 2004; Bennetzen et al., 2005; Grover and Wendel, 2010). Unequal intrastrand recombination occurs between adjacent direct repeats and requires large regions of homology, whereas illegitimate recombination can result from several different mechanisms without the requirement of large regions of homology (Bennetzen, 2002). The direct long terminal repeats at the two ends of LTR elements make them perfect targets for unequal intrastrand recombination, and various types of deletion could happen within and between LTR elements (Devos et al., 2002). Solo LTRs are the direct results from some types of deletions caused by unequal intrastrand recombination as demonstrated in Devos et al. (2002), and they provide evidence of the occurrence of unequal intrastrand recombination. Indeed, solo LTRs do exist in both two genomes, *Ai* and *Ad*, with the ratios of LTR elements to intact LTR retrotransposons being greater than 2 in inverted regions (**Table 3**). However, the ratios of LTR elements to intact LTR retrotransposons in the inverted regions in *Ad* are not consistently greater than in the corresponding regions in *Ai*, and those ratios are quite similar between two species – suggesting that unequal intrastrand recombination doesn’t account for the majority of genome size reduction in those inverted regions in *Ad* (**Table 3**). Because occurrence of unequal intrastrand recombination requires homology and illegitimate recombination does not, we can examine the unaligned sequences discussed earlier, to help categorized deletions possibly caused by illegitimate recombination and unequal intrastrand recombination. Since homologous sequences of at least 50 bp in length are sufficient for homologous recombination (including unequal intrastrand recombination) in yeast (Sugawara and Haber, 1992), we excluded those repeats shorter than 50 bp in the analysis of flanking repeats. Out of 1,903 unaligned sequences longer than 1,000 bp in *Ai*, 83 (4.36%) have two identical repeats (match to the same repeat in RepeatMasker) in the same direction at the two ends of the unaligned sequence, indicating that these unaligned sequences are possibly deletions in *Ad* caused by unequal intrastrand recombination. 195 out of 1,903 (10.25%) unaligned sequences have two similar repeats (match to repeats from the same class/family in RepeatMasker) in the same direction at the two ends, which still leaves a large portion of unaligned sequences unexplained. For the total length of unaligned sequences longer than 1,000 bp in *Ai*, 2.29% are covered by paired identical repeats and 10.66% are covered by paired similar repeats – in both cases, with the repeats being in the same direction at the two ends. These results together demonstrate that unequal intrastrand recombination is not the major mechanism behind the genome size reduction in the inverted regions in *Ad* – suggesting that illegitimate recombination is the dominant factor.

**Table 3:** Solo and intact LTRs in inverted regions in *Ai* and *Ad.*

*Ai*					*Ad*				
Chrom	Position	No. LTRs	No. solo LTRs	Solo LTRs /LTRs	Chrom	Position	No. LTRs	No. solo LTRs	Solo LTRs /LTRs
B01	1–17,229,197	243	563	2.32	A01	1–12,027,097	95	246	2.59
B01	110,305,867 –end^a^	651	1,828	2.81	A01	89,417,045 –end^a^	151	435	2.88
B05	104,400,112 –145,654,815	1,107	3,533	3.19	A05	81,408,196 –104,831,952	249	708	2.84
B06	1–27,321,949	1,328	3,077	2.32	A06	1–1,880,291	237	701	2.96
B09	131,019,283 –end^a^	221	542	2.45	A09	108,980,673 –end^a^	89	213	2.39

*^a^ “end” represents the end position of that chromosome.*

### Relative Rate of Size Reduction in Inverted Regions

The inversions and the following genome size changes observed between *Ai* and *Ad* provide an unusual opportunity to investigate the rate of size reduction after the inversion. Because deletions, insertions, duplications and other kinds of processes affecting the genome size took place simultaneously during the evolution, the rate of size reduction measured here is the net rate without distinguishing the effects of different processes. Assuming all the inversions occurred immediately after the divergence of the two *Arachis* species, the inversions would have occurred about 2.16 million years ago, based on estimated divergence estimates in Bertioli et al. (2016). The inverted regions have decreased to 84 Mbp in *Ad*, relative to 129 Mbp for those regions in *Ai*, in 2.16 million years. With the assumption of one generation per year, the relative rate of size reduction of inverted regions is 2.0 × 10^−5^ bp per 100 base pairs per generation. In other words, the inverted regions in *Ad* lost approximately 26 bases every generation in that 129 Mbp assuming one generation per year. Although both of the two *Arachis* species are annual in their native environment (Krapovickas et al., 2007; Samoluk et al., 2015), the generation rate may be less than yearly considering several environmental factors. One generation every 2 years would double the rate of size reduction to 4.0 × 10^−5^ bp per 100 base pairs per generation. The relative rates of size reduction are not identical for all those inversions. The longest inversion (on chromosome 5) has the highest rate, at 2.6 × 10^−5^ bp per 100 base pairs per generation, and the shortest inversion (on chromosome 9) has the lowest rate, at 1.5 × 10^−5^ bp per 100 base pairs per generation, assuming one generation per year. This suggests that larger inversions lead to larger changes in recombination rates after the inversion, and more rapid size reduction in the inverted region. It is possible, and most likely, that not all inversions occurred right after the divergence of the two *Arachis* species, and this would make the relative rate of reduction even higher for those younger inversions. It is also possible that sequence removal was uneven and the losses may have occurred most rapidly in the early generations.

### Tests of the Model in Other Species

The model of genome size reduction discussed above was suggested based on the observations and studies between two species: *Ai* and *Ad*. Are similar mechanisms at play in other plant species? To test our model, we looked for instances of clear inversions between two species, where it was possible to determine the ancestral genomic orientations (and the affected species of the inversion) by comparisons with genomes from other related species. An inversion is found between *Phaseolus vulgaris* (common bean) chromosome 8 and *Vigna radiata* (mung bean) chromosome 6, and the comparison between *Glycine max* (soybean) and *Vigna radiata* indicates that the inversion likely happened in *Phaseolus vulgaris* (**Supplementary Figures 3A,B**). In the inverted region, size ratios of syntenic blocks follow the pattern predicted by our model of genome size reduction, with formerly proximal region getting smaller after becoming distal, and the formerly distal region getting larger after becoming proximal (**Supplementary Figure 3C**). Another inversion is evident between *Sorghum bicolor* (sorghum) chromosome 4 and *Setaria italica* (foxtail millet) chromosome 1; and a comparison between *Brachypodium distachyon* (purple false brome) and *Sorghum bicolor* suggests that the inversion occurred in *Setaria italica* (**Supplementary Figures 4A,B**). Again, size ratios of syntenic blocks in the inverted region show the same pattern as our model of genome size reduction (**Supplementary Figure 4C**). There is also a larger inversion between *Zea mays* (maize) chromosome 3 and *Sorghum bicolor* chromosome 3, and comparison with *Brachypodium distachyon* suggests that the inversion took place in *Zea mays* (**Supplementary Figures 5A,B**). However, in this case, almost every syntenic block in *Zea mays* is larger than the corresponding syntenic block in *Sorghum bicolor*. We speculate that this is due to the highly proliferated TEs throughout the *Zea mays* genome (Schnable et al., 2009). This explanation is supported by the fact that size ratios of syntenic blocks in newly distal region (formerly proximal) are smaller than the size ratios of syntenic blocks in newly proximal region (formerly distal) – indicating that the mechanism of genome size reduction we proposed still plays an important role in this situation (**Supplementary Figure 6**). These results demonstrate that the model of genome size reduction applies to not only the two *Arachis* species but also other plant species.

### Generalization to Other Types of Genomic Rearrangements

We show that large inversions have the ability to cause substantial sequence loss in the inverted region due to the exposure of formerly proximal (pericentromeric) region to high recombination rate as they move into distal (telomeric) environments. However, inversions are probably not the only type of genomic rearrangement which can cause genome size reduction in affected regions. Other types of genomic rearrangement such as translocations and chromosome breakages, which also expose proximal regions to high recombination rates, can also lead to genome size reductions in the affected regions. Chromosomes 7 and 8 in these two *Arachis* species have experienced several genomic rearrangements (**Figure 3**). Studies have shown that the small pair of “A” chromosomes (pseudomolecule Aradu.A08 = cytogenetic A09) is a characteristic derived state of the A-genome species (Krapovickas et al., 2007; Robledo et al., 2009; Moretzsohn et al., 2013; Bertioli et al., 2016). We maintain that the *Ai* chromosomes have retained the ancestral state, with normal distribution of gene densities (**Figure 2**), while in *Ad*, chromosomes 7 and 8 broke into nine pieces and recombined to form two reconstituted chromosomes 7 and 8 – which subsequently shrank twofold overall [(*Ad*07 + *Ad*08)/(*Ai*07 + *Ai*08) = 0.50], due to exposure of formerly proximal material to a new distal environment following the rearrangements. In the model in **Figure 3**, the second segment in light green on B08 got smaller after becoming distal in A07. The second segment in light blue on B07 also got smaller after becoming more distal in A07 (comparing the relative position on the chromosome) (**Figure 3**). Other regions retained their respective genomic positions (distal or proximal), and accordingly, remained approximately the same sizes in *Ai* and *Ad*. These more static regions include the orange and dark green segments in **Figure 3**, which are distal in both *Ai* and *Ad*.

## Discussion

Our study shows that the inversions occurring in *Ad* after speciation with *Ai* led to subsequent genome size reduction through the net genomic sequence loss in inverted regions. TEs are the main sources of sequence loss in the inverted regions in *Ad*. Illegitimate recombination is likely the primary mechanism causing the sequence deletions rather than unequal intrastrand recombination. The net sequence loss is due to more rapid sequence losses at the newly distal end, compared with much slower sequence gains at the newly proximal end. This means that the locational difference in sequence losses and gains is mainly responsible for the genome size reduction. Our results indicate that the chromosomal location determines the recombination rate, which is the major driver behind the sequence loss processes. Thus, relocating a genomic segment to a different chromosomal environment changes the landscape of recombination, which then affects the sequence losses and gains within that genomic segment.

Our results indicate that only a small proportion of the deletions in *Ad* can be attributed to unequal intrastrand recombination, with most of the remaining deletions likely being due to illegitimate recombination. This observation is consistent with the previous studies in *Arabidopsis* and rice showing that illegitimate recombination is the primary mechanism responsible for DNA removal (Devos et al., 2002; Ma et al., 2004). Although there is a dramatic removal of LTR retrotransposons in the inverted regions in *Ad*, it is not because those LTR retrotransposons serve as the homologous regions initiating unequal intrastrand recombination. It is possibly due to the DNA content removal in LTR retrotransposon enriched intergenic regions, since removing DNA content from intragenic regions is more likely to have deleterious effects and not able to survive the natural selection.

A remaining question about this genome size reduction process is to what extent selection plays a role in the process. Our model of genome size reduction doesn’t require selection, but it is possible that selection pressure has an effect on the process. If there is selective benefit to a smaller genome, size reductions would be favored for fixation in a population. Studies have shown that several physiological and ecological traits are associated with genome size (Sparrow and Miksche, 1961; Ceccarelli et al., 1993; Wakamiya et al., 1993; Greilhuber and Obermayer, 1997; Chung et al., 1998; Beaulieu et al., 2007; Samoluk et al., 2015). However, it is still not established whether the natural selection generally favors or acts against a smaller genome (Bennetzen, 2002). Although both unequal intrastrand recombination and illegitimate recombination don’t need selection pressure to remove DNA content, there are mechanisms such as unequal interstrand recombination that require selection to remove DNA content. Unequal interstrand recombination will generate reciprocal deletion and insertion on the two strands, and selection influences whether the deletion or the insertion is retained. It is hard to tell whether selection plays an important role in the genome size reduction based on the data in this study. Resequencing data from a population would help us understand this question better by looking at the frequency of deletion polymorphisms in the population.

The inversions occurring in one of the two sequenced *Arachis* diploid species provide a valuable opportunity to observe and measure the effects of genomic rearrangements on genome size evolution. The results indicate that the recombinational rates, determined by chromosomal location, are the major driver promoting the sequence removal in the inverted regions. Further, sequence removal is a more potent process in large inversions than TE insertion (at least on the timescale of several million years), leading to the size reduction of the inverted regions. In fact, any type of genomic rearrangement that exposes proximal regions to higher recombination rates (e.g., breakage or translocation) are also able to cause the genome size reduction. This model of genome size reduction is general. We observe the model to hold not only in *Arachis* species, where we first noticed the pattern, but in several other plant genomes (monocot and dicot) tested in this paper. Indeed, the model should hold for any genome in which the following conditions are present: higher rates of recombination near chromosome ends, abundant non-genic material available for removal following genomic rearrangements, and ongoing transposon activity to gradually build up transposon densities in proximal/pericentromeric regions.

## Author Contributions

SC and DB developed the idea and the model. LR performed most of the analysis. SC generated the dot-plots. EC generated the chromosome view of gene densities and recombination rates. WH calculated the synteny between *Ad* and *Ai*. LR and SC wrote and edited the manuscript.

## Conflict of Interest Statement

The authors declare that the research was conducted in the absence of any commercial or financial relationships that could be construed as a potential conflict of interest.

## Footnotes

1 https://peanutbase.org/

2 https://legumeinfo.org/

3 https://phytozome.jgi.doe.gov/pz/portal.html

4 http://www.repeatmasker.org/
