# Supplementary material for: A Mechanism for Genome Size Reduction Following Genomic Rearrangements
Source: Front Genet. 2018 Oct 9;9:454. doi: 10.3389/fgene.2018.00454 (PMC6189423; doi:10.3389/fgene.2018.00454)
Supplement: Supplementary file 2 [file Data_Sheet_1.PDF]

Supplementary Material

A Mechanism for Genome Size Reduction Following Genomic Rearrangements

Longhui Ren, Wei Huang, Ethalinda K. S. Cannon, David J. Bertioli, Steven B. Cannon*

*** Correspondence:** Steven B. Cannon: Steven.Cannon@ARS.USDA.GOV

1 Supplementary Tables and Figure

1.1 Supplementary Tables

Supplemental Table 1. R^2 between TE/gene ratios and size ratios of corresponding syntenic blocks

Chromosome pair[a]	TE ratios vs. Size ratios	Gene ratios vs. Size ratios
1[b]	0.90	0.11
2	0.90	0.28
3	0.94	0.24
4	0.80	0.14
5[b]	0.95	0.15
6[b]	0.95	0.29
7	0.95	0.13
8	0.90	0.22
9[b]	0.94	0.27
10	0.84	0.08

[a] Chromosome pair is formed by the corresponding chromosomes in two genomes. For example, A01 and B01 form the chromosome pair 1.

[b] This chromosome pair has inversion(s) between the two corresponding chromosomes.

1.2 Supplementary Figures

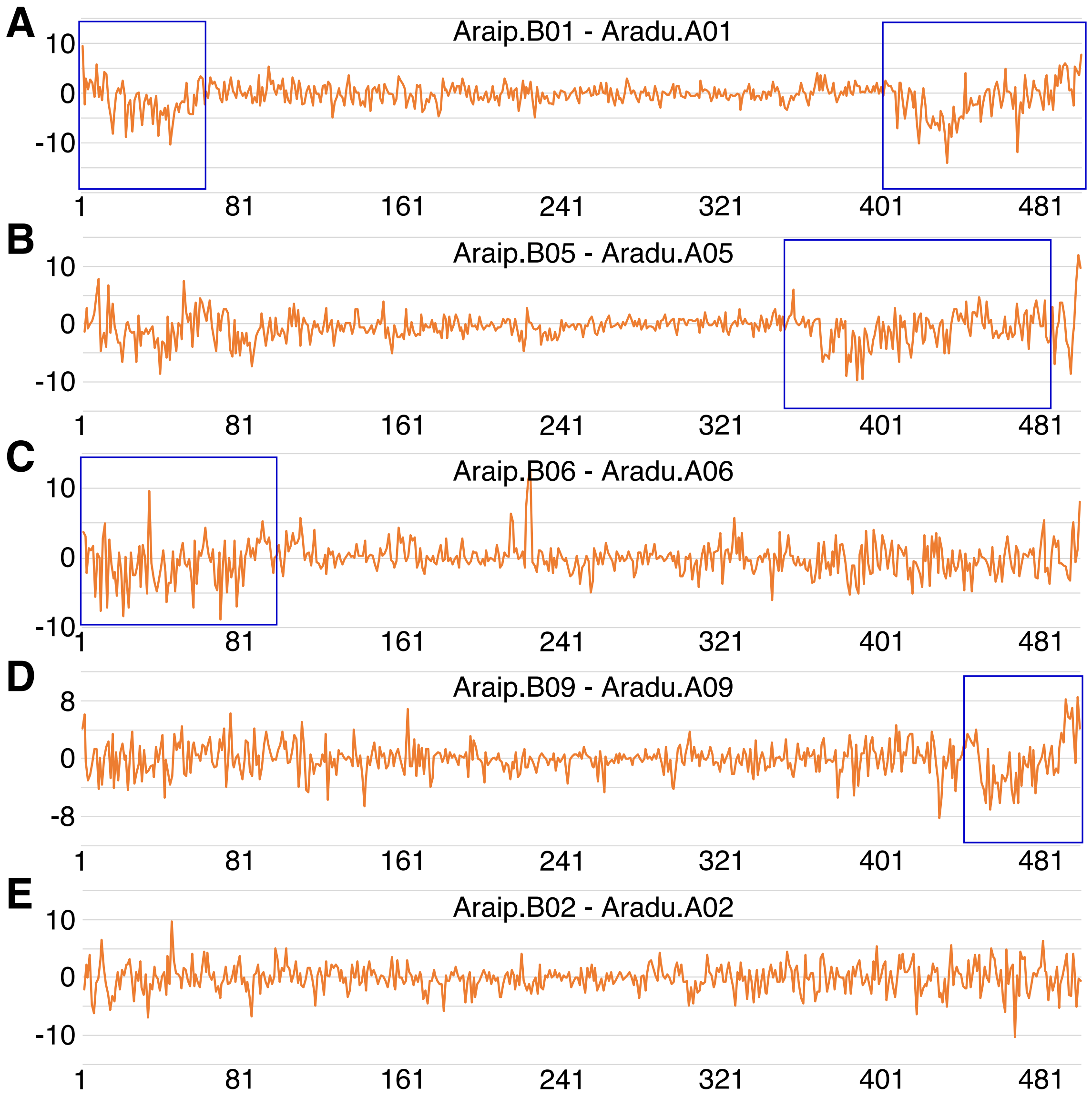

Supplemental Figure 1 Gene density differences along the chromosome between *Ai* and *Ad*. X-axis is the index of 500 partitions along the chromosome, and Y-axis is the gene density difference of each partition. Regions in the blue rectangles are the inverted regions between two *Arachis* species. Inverted regions show unusual density difference with more positive differences at the distal end and more negative differences at the proximal end. **(A)(B)(C)(D)(E)** Gene density differences along chromosome. The chromosomes being compared are indicated on each graph. **(A)(B)(C)(D)** show chromosomes with inversions, whereas **(E)** shows chromosome without inversion.

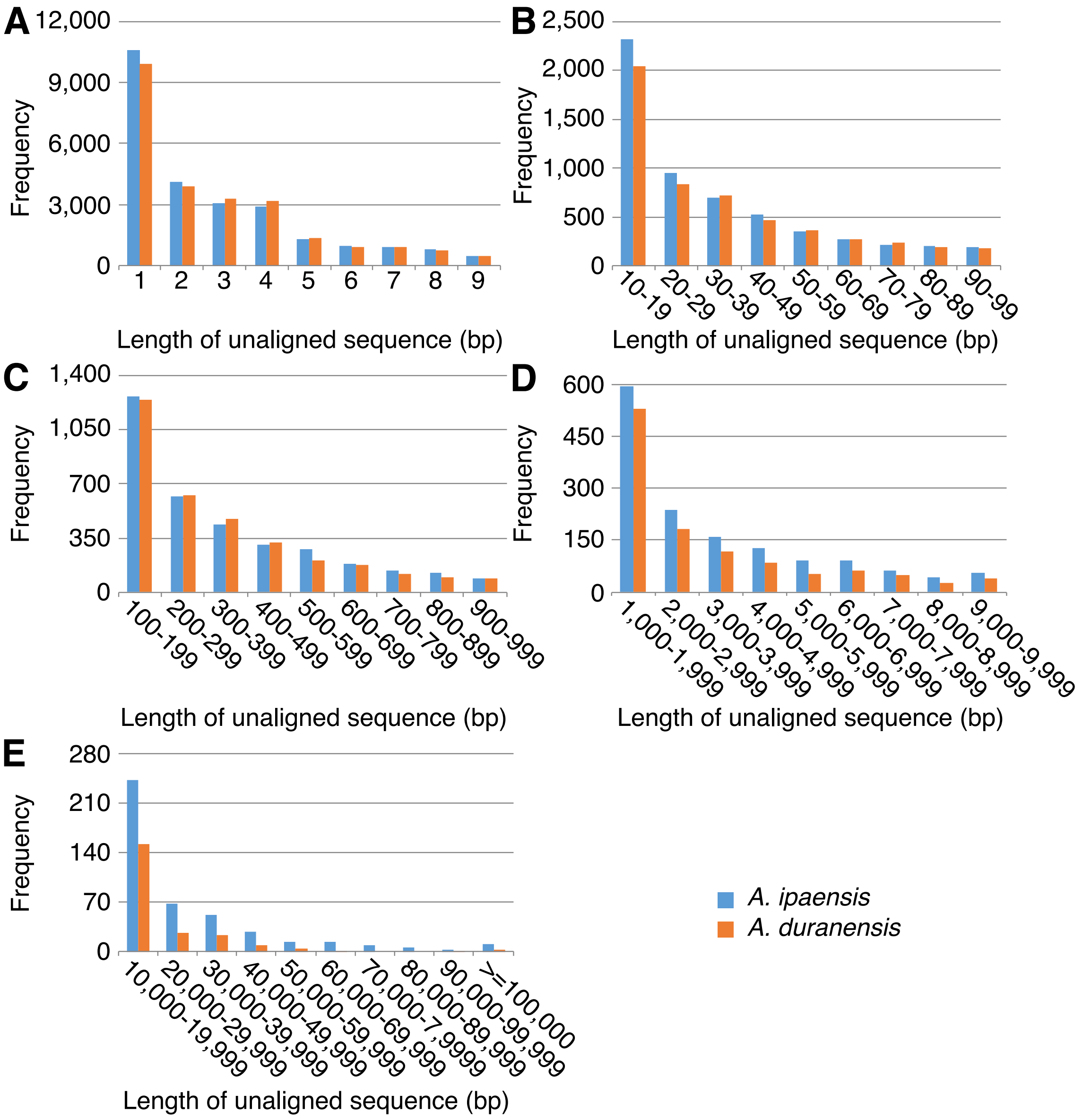

Supplemental Figure 2 Number of unaligned sequences in different length intervals. X-axis is the length intervals of unaligned sequences, and Y-axis is the number of unaligned sequences. Blue bars represent number of unaligned sequences in *Ai*, whereas orange bars represent that in *Ad*. **(A)** Number of unaligned sequences in length intervals from 1 to 9 bp. **(B)** Number of unaligned sequences in length intervals from 10 to 99 bp. **(C)** Number of unaligned sequences in length intervals from 100 to 999 bp. **(D)** Number of unaligned sequences in length intervals from 1,000 to 9,999 bp. **(E)** Number of unaligned sequences in length intervals from 10,000 bp to longer.

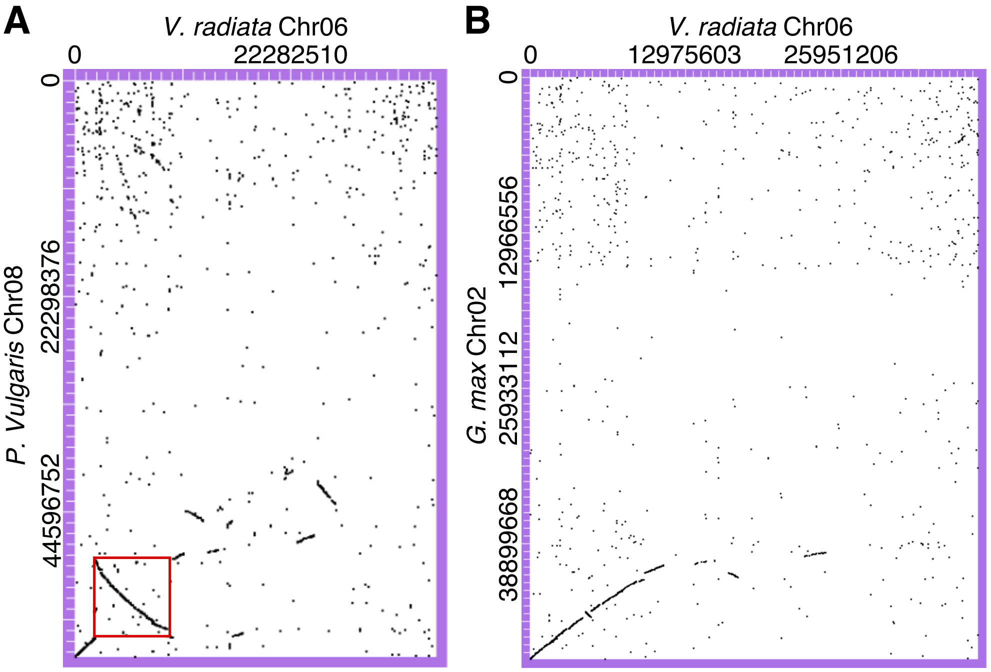

C

Molecue_1	Start_Mol1	End_Mol1	Direction	Molecue_2	Start_Mol2	End_Mol2	Size_Mol1	Size_Mol2	Size_Mol1/Size_Mol2
Vr06	6953	2072388	-	Pv08	57594597	59603568	2065435	2008971	1.028105931
Vr06	2215784	2537527	+	Pv08	49929870	50530324	321743	600454	0.535832886
Vr06	2589623	6001123	+	Pv08	50847303	54218006	3411500	3370703	1.01210341
Vr06	6065536	6241762	+	Pv08	54452569	54601764	176226	149195	1.181178994
Vr06	6260869	6386330	-	Pv08	49803560	49909203	125461	105643	1.187594067
Vr06	6454477	8884693	+	Pv08	54652259	56500015	2430216	1847756	1.315225603
Vr06	9112585	9492959	+	Pv08	56538214	56690206	380374	151992	2.502592242
Vr06	9563098	10059643	+	Pv08	57434235	57592056	496545	157821	3.146254301
Vr06	10071923	11161014	-	Pv08	48942117	49506394	1089091	564277	1.93006449
Vr06	12383551	13257511	+	Pv08	44781332	45503846	873960	722514	1.209609779
Vr06	13798514	14003994	-	Pv08	48684565	48787819	205480	103254	1.990043969
Vr06	14482565	14718672	-	Pv08	48502565	48620046	236107	117481	2.009746257
Vr06	15716522	16266535	-	Pv08	45571329	46010744	550013	439415	1.251693729
Vr06	16553521	17338606	-	Pv08	57150333	57415762	785085	265429	2.957796624
Vr06	22391657	22478085	+	Pv08	40326757	40459309	86428	132552	0.652030901
Vr06	22955024	24687082	-	Pv08	47082121	47826430	1732058	744309	2.327068462
Vr06	25174103	25632175	+	Pv08	41788503	42195832	458072	407329	1.124574975
Vr06	25698777	26384317	+	Pv08	42454760	43089999	685540	635239	1.07918437
Vr06	26562231	26932460	+	Pv08	43401090	43798111	370229	397021	0.932517424

Largest — 1 — Smallest

Supplemental Figure 3 Test of genome size reduction model between *Phaseolus vulgaris* and *Vigna radiata*. **(A)** Dot-plot comparison between *P. vulgaris* chromosome 8 and *V. radiata* chromosome 6 reveals an inversion which is highlighted by red rectangle. **(B)** Dot-plot comparison between *V. radiata* chromosome 6 and *Glycine max* chromosome 2 suggests that the inversion occurred in *P. vulgaris*. **(C)** Size ratios of syntenic blocks between *P. vulgaris* chromosome 8 and *V. radiata* chromosome 6. Inverted region is highlighted by red rectangle. The size ratios are calculated as *V. radiata*/*P. vulgaris*, and are shown in the blue-white-red gradient.

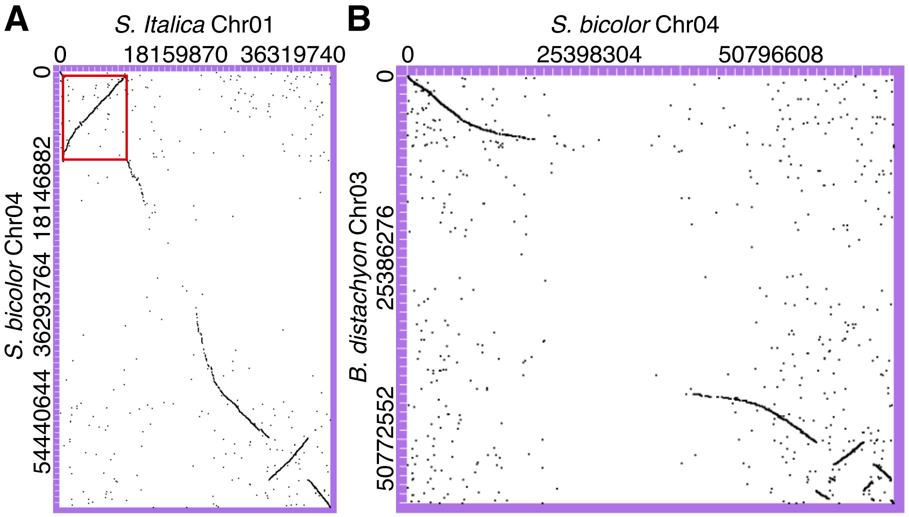

C

Molecue_1	Start_Mol1	End_Mol1	Direction	Molecue_2	Start_Mol2	End_Mol2	Size_Mol1	Size_Mol2	Size_Mol1/Size_Mol2
Sb04	64623	687161	+	Si01	74818	490951	622538	416133	1.496007286
Sb04	205875	412250	+	Si01	157892	294826	206375	136934	1.507112916
Sb04	763385	1077810	+	Si01	9983376	10301204	314425	317828	0.989292951
Sb04	1083083	7877044	-	Si01	3655618	9894409	6793961	6238791	1.088986792
Sb04	7885119	8356666	+	Si01	3107193	3436947	471547	329754	1.4299963
Sb04	8454382	11803579	-	Si01	1096291	3080386	3349197	1984095	1.688022499
Sb04	12059009	12543232	-	Si01	676263	1048325	484223	372062	1.301457822
Sb04	12822620	12869693	-	Si01	620929	648235	47073	27306	1.723906834
Sb04	13378885	13706802	-	Si01	573180	622047	327917	48867	6.71039761
Sb04	13843377	14243963	+	Si01	10428890	10769882	400586	340992	1.174766563
Sb04	14597479	14978971	+	Si01	10766511	10917750	381492	151239	2.522444608
Sb04	16302954	16373950	-	Si01	11294259	11379761	70996	85502	0.83034315
Sb04	16409546	16514499	+	Si01	11615866	11791489	104953	175623	0.597603958
Sb04	16515898	16913556	-	Si01	11970003	12254767	397658	284764	1.396447585
Sb04	18848973	19229334	+	Si01	12782071	12902735	380361	120664	3.152232646
Sb04	37657457	37913163	+	Si01	21253152	21330638	255706	77486	3.300028392
Sb04	40849571	41069092	+	Si01	21991841	22048505	219521	56664	3.87408231
Sb04	41530572	42173412	+	Si01	22070869	22163345	642840	92476	6.951425235
Sb04	44100636	44405463	+	Si01	22607214	22684599	304827	77385	3.939096724
Sb04	44640699	45091312	+	Si01	22769774	22861969	450613	92195	4.887607788
Sb04	45674769	45989763	+	Si01	22993688	23230264	314994	236576	1.331470648
Sb04	47153270	47450242	+	Si01	23754885	23898192	296972	143307	2.072278395
Sb04	47677816	47947242	+	Si01	23896304	24014949	269426	118645	2.270858443
Sb04	48182254	48844142	+	Si01	24022328	24451081	661888	428753	1.543751297
Sb04	49108659	49357490	+	Si01	24617638	24793269	248831	175631	1.416782914
Sb04	49598532	55720025	+	Si01	24902201	30832587	6121493	5930386	1.032225052
Sb04	55786851	57072488	+	Si01	31244121	32524276	1285637	1280155	1.004282294
Sb04	57095152	63670632	-	Si01	32531233	38630122	6575480	6098889	1.078143905
Sb04	63682026	67960074	+	Si01	38766840	42061795	4278048	3294955	1.298363104

Largest — 1 — Smallest

Supplemental Figure 4 Test of genome size reduction model between *Sorghum bicolor* and *Setaria italica*. **(A)** Dot-plot comparison between *S. bicolor* chromosome 4 and *S. italica* chromosome 1 reveals an inversion which is highlighted by red rectangle. **(B)** Dot-plot comparison between *S. bicolor* chromosome 4 and *Brachypodium distachyon* chromosome 3 suggests that the inversion occurred in *S. italica*. **(C)** Size ratios of syntenic blocks between *S. bicolor* chromosome 4 and *S. italica* chromosome 1. Inverted region is highlighted by red rectangle. The size ratios are calculated as *S. bicolor*/*S. italica*, and are shown in the blue-white-red gradient.

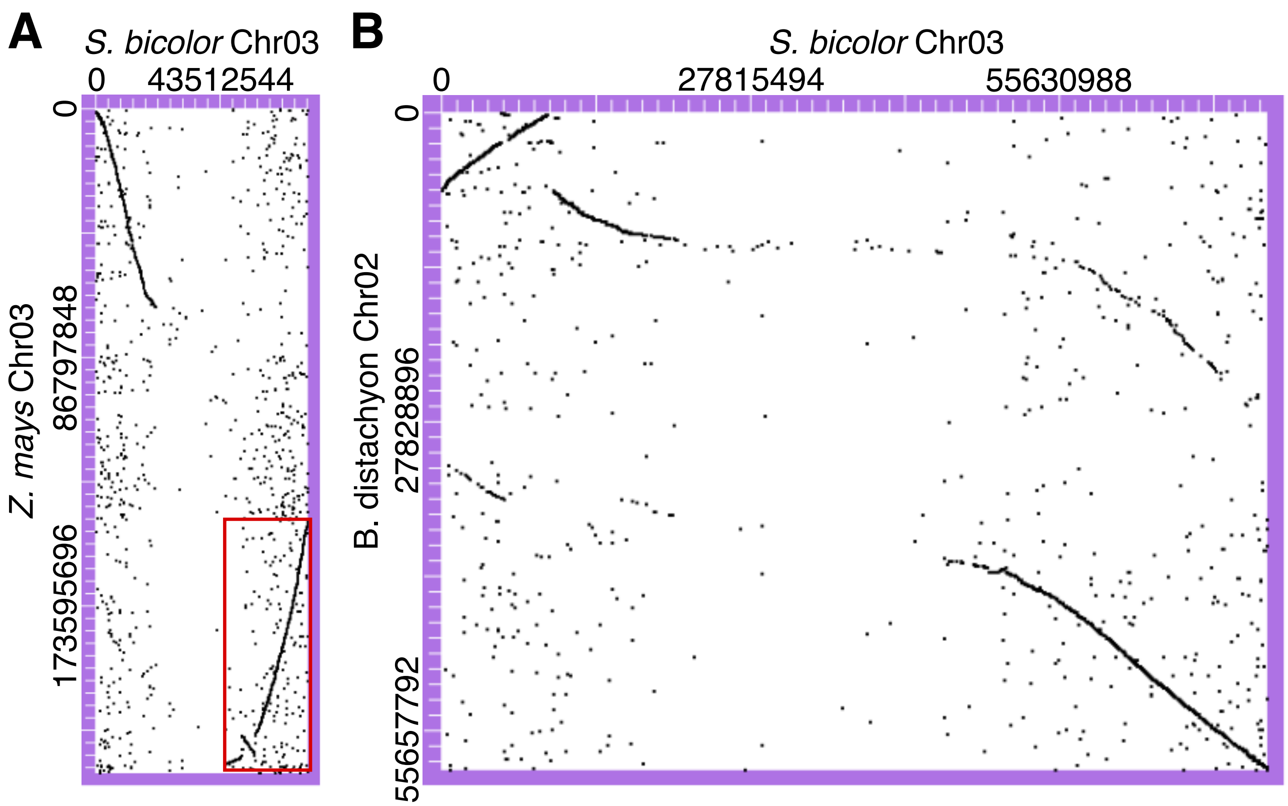

Supplemental Figure 5 Dot-plot comparisons indicate an inversion occurred in *Zea mays*. **(A)** Dot-plot comparison between *Z. mays* chromosome 3 and *Sorghum bicolor* chromosome 3 reveals an inversion which is highlighted by red rectangle. **(B)** Dot-plot comparison between *S. bicolor* chromosome 3 and *Brachypodium distachyon* chromosome 2 suggests that the inversion occurred in *Z. mays*.

Molecue_1	Start_Mol1	End_Mol1	Direction	Molecue_2	Start_Mol2	End_Mol2	Size_Mol1	Size_Mol2	Size_Mol1/Size_Mol2
Sb03	860459	2120414	+	Zm03	2339355	4345261	1259955	2005906	0.628122654
Sb03	1595522	1814858	-	Zm03	3399296	3560593	219336	161297	1.359826903
Sb03	2155646	2245271	-	Zm03	5287322	5438197	89625	150875	0.594034797
Sb03	2326290	2619910	-	Zm03	4345679	4896155	293620	550476	0.533392918
Sb03	2696880	3402232	+	Zm03	5567042	7675155	705352	2108113	0.334589275
Sb03	3458565	3557660	+	Zm03	7970386	8304232	99095	333846	0.296828478
Sb03	3562688	4394778	+	Zm03	8536278	11013400	832090	2477122	0.335909979
Sb03	4471209	4557470	+	Zm03	11403408	11660545	86261	257137	0.335467086
Sb03	4568874	4792583	+	Zm03	12031159	12890853	223709	859694	0.260219334
Sb03	4831164	5160880	+	Zm03	13212794	14121103	329716	908309	0.362999816
Sb03	5224030	5594503	+	Zm03	14693148	15482123	370473	788975	0.469562407
Sb03	5690813	5759026	+	Zm03	16476743	16655507	68213	178764	0.381581303
Sb03	5807178	6407589	+	Zm03	17028296	18240944	600411	1212648	0.495123894
Sb03	6484814	6661548	+	Zm03	18826726	19470533	176734	643807	0.274513946
Sb03	7104195	7663632	+	Zm03	21151937	23847768	559437	2695831	0.207519314
Sb03	7956628	8116789	+	Zm03	25124314	25354228	160161	229914	0.696612646
Sb03	8143749	8426938	+	Zm03	25862616	26454329	283189	591713	0.478591817
Sb03	8667001	8713999	+	Zm03	28222060	28402122	46998	180062	0.261010097
Sb03	8746377	8998664	-	Zm03	28820448	29480102	252287	659654	0.382453529
Sb03	9047057	9231824	+	Zm03	29510600	30239339	184767	728739	0.25354345
Sb03	9251521	9322478	+	Zm03	30441422	30571432	70957	130010	0.545781094
Sb03	9331521	9366200	+	Zm03	30853819	31106316	34679	252497	0.137344206
Sb03	9495617	9560920	+	Zm03	31389203	31559126	65303	169923	0.384309364
Sb03	9586443	9894680	+	Zm03	31805298	33129415	308237	1324117	0.232786831
Sb03	9899225	10087497	+	Zm03	33349699	34110359	188272	760660	0.247511372
Sb03	10122556	10217347	+	Zm03	34440861	34681584	94791	240723	0.393776249
Sb03	10296136	10529751	+	Zm03	35353590	36002508	233615	648918	0.360006965
Sb03	10542401	10618992	+	Zm03	36217547	36594287	76591	376740	0.203299358
Sb03	10633982	10740994	+	Zm03	36833446	37361914	107012	528468	0.202494758
Sb03	10780856	10890224	+	Zm03	37667628	38062764	109368	395136	0.276785714
Sb03	10987405	11085099	-	Zm03	40521477	40624693	97694	103216	0.946500543
Sb03	11161438	11322283	-	Zm03	39211508	39480438	160845	268930	0.59809244
Sb03	11362563	11416948	-	Zm03	38438235	38747707	54385	309472	0.1757348
Sb03	11658360	11892299	-	Zm03	41244193	41744737	233939	500544	0.467369502
Sb03	11974696	12126543	+	Zm03	43359914	43661620	151847	301706	0.503294598
Sb03	12156620	12320533	+	Zm03	43867499	44679224	163913	811725	0.201931689
Sb03	12323307	12628921	+	Zm03	45035844	45712949	305614	677105	0.451353926
Sb03	12728659	12956888	+	Zm03	47450743	48109584	228229	658841	0.346409832
Sb03	13107760	13382544	+	Zm03	48795411	49233319	274784	437908	0.627492533
Sb03	14147425	14211171	+	Zm03	52245017	52454327	63746	209310	0.304553055
Sb03	14652639	14828121	+	Zm03	53883227	54254798	175482	371571	0.472270441
Sb03	14964590	15056554	+	Zm03	54699621	55172779	91964	473158	0.194362137
Sb03	15056859	15430025	+	Zm03	55375451	56017264	373166	641813	0.581424808
Sb03	15676108	15807065	+	Zm03	56813678	57231705	130957	418027	0.313274023
Sb03	15953739	16065394	+	Zm03	58715704	59047011	111655	331307	0.337013706
Sb03	16406571	16735073	+	Zm03	60531549	60951573	328502	420024	0.782102927
Sb03	47886152	48077579	-	Zm03	227582227	227862209	191427	279982	0.68371181
Sb03	48828110	49118685	-	Zm03	227312894	227514555	290575	201661	1.440908257
Sb03	49614386	49797467	-	Zm03	227133973	227282452	183081	148479	1.233043057
Sb03	50312420	50904453	-	Zm03	226221276	227047835	592033	826559	0.716262239
Sb03	51021293	51097607	-	Zm03	225910755	226006102	76314	95347	0.800381763
Sb03	51298718	51446229	+	Zm03	219181047	219342672	147511	161625	0.912674401
Sb03	51666701	53396767	+	Zm03	219454655	221791498	1730066	2336843	0.740343275
Sb03	53434350	53520839	+	Zm03	221999802	222144826	86489	145024	0.596377151
Sb03	53525192	54425379	+	Zm03	222563324	223769272	900187	1205948	0.7464559
Sb03	54629984	55316480	+	Zm03	223769796	224719118	686496	949322	0.723143464
Sb03	55375373	55661845	+	Zm03	225003062	225848000	286472	844938	0.339044995
Sb03	55736569	57877155	-	Zm03	214256438	218631182	2140586	4374744	0.489305431
Sb03	59317050	59697672	-	Zm03	208411907	209221093	380622	809186	0.470376403
Sb03	59719038	60824658	-	Zm03	205063871	208194489	1105620	3130618	0.353163497
Sb03	60827256	61393355	-	Zm03	202838553	204791875	566099	1953322	0.289813456
Sb03	61464172	61956033	-	Zm03	200770762	202064951	491861	1294189	0.380053454
Sb03	62033089	62569872	-	Zm03	199004703	200505886	536783	1501183	0.357573327
Sb03	62582974	62629884	+	Zm03	196788699	197089273	46910	300574	0.156068056
Sb03	62654858	62998604	+	Zm03	197317334	198990186	343746	1672852	0.205485004
Sb03	62999578	63662222	-	Zm03	194319036	196668478	662644	2349442	0.28204314
Sb03	63676144	64017152	-	Zm03	192784558	194118681	341008	1334123	0.255604618
Sb03	64075421	64777193	-	Zm03	190373044	192298354	701772	1925310	0.364498185
Sb03	65055257	65999368	-	Zm03	185736397	188988435	944111	3252038	0.290313643
Sb03	66001934	66068100	-	Zm03	185302662	185493650	66166	190988	0.346440614
Sb03	66078929	66439792	-	Zm03	183899079	185090499	360863	1191420	0.302884793
Sb03	66443112	67242161	-	Zm03	180553262	183703536	799049	3150274	0.253644286
Sb03	67266364	67934495	-	Zm03	178053577	180312482	668131	2258905	0.295776493
Sb03	67948918	68004428	-	Zm03	177490009	177843405	55510	353396	0.157075915
Sb03	68119874	68285961	-	Zm03	176302414	176862127	166087	559713	0.296736006
Sb03	68294922	68604252	-	Zm03	175226380	176089875	309330	863495	0.358230216
Sb03	68609510	68760814	-	Zm03	174328423	174877966	151304	549543	0.275326953
Sb03	68789093	68932692	-	Zm03	173297514	173846615	143599	549101	0.261516552
Sb03	68937532	69286996	-	Zm03	171471054	173066203	349464	1595149	0.219079221
Sb03	69290487	69660043	-	Zm03	169620144	171199550	369556	1579406	0.233984169
Sb03	69855029	69959621	-	Zm03	168275428	168569728	104592	294300	0.355392457
Sb03	70006727	70068297	-	Zm03	167405942	167531278	61570	125336	0.491239548
Sb03	70095150	70572572	-	Zm03	165488281	167118128	477422	1629847	0.292924428
Sb03	70640219	70729867	-	Zm03	164036105	164631995	89648	595890	0.150443874
Sb03	70758314	71141978	-	Zm03	161295516	162874516	383664	1579000	0.242979101
Sb03	71146045	71247025	-	Zm03	160346411	160978909	100980	632498	0.159652679
Sb03	71286653	71392952	-	Zm03	159720230	160078095	106299	357865	0.297036592
Sb03	71457464	71517339	-	Zm03	158932008	159050063	59875	118055	0.507178857
Sb03	71579203	71674560	-	Zm03	158390857	158613255	95357	222398	0.428767345
Sb03	71698807	71767123	-	Zm03	157455519	157799784	68316	344265	0.198440155
Sb03	71805307	71986902	-	Zm03	156317527	157250798	181595	933271	0.194579067
Sb03	71995842	72239128	-	Zm03	155358894	156113239	243286	754345	0.322512909
Sb03	72285158	72377425	-	Zm03	154450608	154782086	92267	331478	0.278350298
Sb03	72396094	72536796	-	Zm03	153802447	154165422	140702	362975	0.387635512
Sb03	72682883	72747268	-	Zm03	151657877	152013220	64385	355343	0.181191131
Sb03	72771920	72862098	-	Zm03	150760194	151397210	90178	637016	0.141563163
Sb03	72863517	72902017	-	Zm03	150088574	150311198	38500	222624	0.172937329
Sb03	72989525	73082877	-	Zm03	149268271	149604353	93352	336082	0.277765545
Sb03	73162025	73477084	-	Zm03	147446876	148596450	315059	1149574	0.274065871
Sb03	73564683	74119532	-	Zm03	145209829	147054061	554849	1844232	0.3008564
Sb03	74324766	74358386	-	Zm03	144109896	144134374	33620	24478	1.373478225

Largest 1 Smallest

Supplemental Figure 6 Size ratios of syntenic blocks between *Zea mays* chromosome 3 and *Sorghum bicolor* chromosome 3. Inverted region is highlighted by blue rectangle. The size ratios are calculated as *S. bicolor*/*Z. mays*, and are shown in the blue-white-red gradient.
